# Lung cancer screening: an urgent necessity

**DOI:** 10.36416/1806-3756/e20250344

**Published:** 2025-09-22

**Authors:** Gustavo Faibischew Prado, Thiago Lins Fagundes de Sousa, Isabele Alves Chirichela

**Affiliations:** 1. Comissão Científica de Câncer, Sociedade Brasileira de Pneumologia e Tisiologia, Brasília (DF) Brasil.; 2. Rede D’Or, São Paulo (SP) Brasil.; 3. Universidade Federal de Campina Grande, Campina Grande (PB) Brasil.

Lung cancer screening (LCS) was brought back to the center of the debate in Brazil after a public hearing on a bill proposing the implementation of LCS in the country. The hearing was held in the House of Representatives on August 19, 2025, and on August 20, the Senate’s Social Affairs Committee approved a proposal to make August National Month of Lung Cancer Awareness and Prevention, an initiative known as *Agosto Branco* (White August). However, despite this timely alignment, health leaders and decision-makers remain adamant, arguing that the Brazilian Unified Health Care System currently struggles to diagnose and treat patients presenting with symptoms or incidental findings, and that adding screening would overburden a network that “cannot meet” the existing demand. 

It is essential to reinforce and expand prevention policies, which have made Brazil a global leader in tobacco control.^1^ It is equally urgent to structure integrated and assertive care pathways for symptomatic patients and patients with incidental imaging findings, thus ensuring swift and definitive routes to diagnosis and treatment. Nevertheless, a significant reduction in lung cancer mortality cannot be achieved without taking further steps. 

The problem is that when symptoms are present, the disease is almost invariably locally advanced or metastatic, and even with an excellent fast-track system the impact on survival and quality of life remains limited. This is not a play on words: “rapid diagnosis of advanced disease is not early diagnosis.” It would be equivalent to abandoning breast cancer screening and waiting for patients to present with palpable masses, ulcerations, or bone pain, or to abandoning cervical cancer screening and intervening only when cases involve genital bleeding or urinary obstruction. 

LCS shifts diagnosis to earlier stages, offering more patients a real chance of cure. For health care systems, LCS also translates to less costly treatments and greater return on investment. Not screening would be akin to treating myocardial infarctions only when patients develop cardiogenic shock, instead of investing in prevention and early diagnosis of coronary disease. Medicine should not wait for a catastrophe to act, and lung cancer should not be an exception. 

It is worth recalling that when a study by the U.S. National Lung Screening Trial Research Team was published in 2011,^2^ many experts were skeptical about LCS.^3^ ^,^ ^4^ Many questioned whether the results would be reproducible in other scenarios; whether benefits would extend to different populations; how to cope with overdiagnosis^5^; and whether it would be justifiable to propose an expensive screening program in countries with profound inequalities in primary health care. In Brazil, skepticism also included concerns about the fact that Brazil is a vast country in which specialized professionals are unevenly distributed.^6^ However, technological advances now allow CT scans to be remotely reviewed by radiologists and specialists anywhere in the country, helping to mitigate barriers related to misdiagnosis and disparities in expertise. Many of the aforementioned questions have therefore been answered. We now have robust evidence of mortality reduction in different scenarios,^7^ ^,^ ^8^ as well as practical experience showing feasibility in the national context, with questions regarding the high prevalence of tuberculosis in Brazil being answered.^9^ ^,^ ^10^ More recently, low-dose CT screening has been shown to be cost-effective in the Brazilian Unified Health Care System.^11^

This is not a matter of choosing screening, prevention (including regulatory measures addressing the increasing use of electronic nicotine delivery systems),^12^ or improving the diagnostic journey: we need all three in a complementary and integrated manner if we truly want to change the landscape of lung cancer in Brazil. Complex, multifactorial problems require comprehensive solutions. Refusing to implement LCS at this point is simply accepting preventable deaths. According to the International Agency for Research on Cancer, lung cancer cases and lung cancer mortality in Brazil will have increased by 65% and 74%, respectively, by the year 2040.^13^

Ultimately, this discussion is not about statistics; it is about the profound human cost of delayed diagnosis. It concerns the lives of people who could still be at home, working, and spending time with their families, as well as those who are diagnosed too late because they were denied the opportunity for early detection. LCS is not just an option; it has become an urgent necessity. To forgo this critical intervention is to abdicate the responsibility of saving lives and betray the promise of a healthier future. 
